# Interleukin-1 Gene Variability and Plasma Levels in Czech Patients with Chronic Periodontitis and Diabetes Mellitus

**DOI:** 10.1155/2019/6802349

**Published:** 2019-01-14

**Authors:** Petra Borilova Linhartova, Hana Poskerova, Marie Tomandlova, Jirina Bartova, Katerina Kankova, Antonin Fassmann, Lydie Izakovicova Holla

**Affiliations:** ^1^Clinic of Stomatology, Institutions Shared with St. Anne's Faculty Hospital, Faculty of Medicine, Masaryk University, Brno 60200, Czech Republic; ^2^Department of Pathophysiology, Faculty of Medicine, Masaryk University, Brno 62500, Czech Republic; ^3^Department of Biochemistry, Faculty of Medicine, Masaryk University, Brno 62500, Czech Republic; ^4^Institute of Clinical and Experimental Dental Medicine, General University Hospital and First Faculty of Medicine, Charles University, Prague 12000, Czech Republic

## Abstract

Recent studies have suggested a bidirectional relationship between chronic periodontitis (CP) and diabetes mellitus (DM). Immunoregulatory factors such as cytokines play an important role in etiopathogenesis of both diseases. The aim of this study was to analyze variability in interleukin-1 (*IL-1*) gene cluster and IL-1*β* plasma levels in patients with CP, DM, and a combination of both diseases. A total of 1016 individuals participating in this case-control study—225 healthy controls, 264 patients with CP, 132 with type 1 diabetes (T1DM), and 395 patients with type 2 diabetes (T2DM)—were genotyped using methods based on polymerase chain reaction for *IL-1* gene polymorphisms (*IL-1A* (−889C/T, rs1800587), *IL-1B* (+3953C/T, rs1143634), and *IL-1RN* (gene for IL-1 receptor antagonist, IL-1RA, 86 bp tandem repeats in intron 2)). Levels of IL-1*β* were measured by Luminex methods in subgroups of controls, CP, T1DM + CP, and T2DM + CP subjects. Although no significant associations were found in the genotype and allele frequencies of *IL-1A* (−889C/T), significant differences in the allele frequencies of *IL-1B* (+3953C/T) were observed between controls and CP patients (*P* < 0.05). In T1DM patients, *IL-1RN*^*∗*^S *“*short” allele and *IL-1RN* 12 genotype were significantly less frequent than those in controls (*P* < 0.01). In haplotype analysis, TTL haplotype decreased the risk of CP development (*P* < 0.01), whereas CCS and CTL haplotypes (*P* < 0.01 and *P* < 0.05) were associated with T1DM. Although IL-1*β* levels were measured significantly higher in mononuclear cells after stimulation by mitogens, HSP70, or selected periodontal bacteria than in unstimulated cells, *IL-1* genotypes did not correlate with circulating IL-1*β* levels. In the Czech population, significant associations between the *IL-1B* polymorphism with CP and the *IL-1RN* variant with T1DM were found. Haplotype analysis suggests that variability in *IL-1* gene cluster may be one of the factors in the CP and T1DM pathogenesis, although single variants of these polymorphisms are not substantial for protein production.

## 1. Introduction

Chronic periodontitis (CP), an inflammatory disease, which is the result of a complex interplay of bacterial infection and host responses, is characterized by the loss of connective tissue attachment, slowly progressing alveolar bone destruction, and, ultimately, loss of teeth. Molecular patterns (PAMPs) of anaerobic Gram-negative bacteria in the subgingival biofilm, among others, such as *Aggregatibacter actinomycetemcomitans*, *Porphyromonas gingivali*s, *Prevotella intermedia*, and *Tannerella forsythia* [1], are recognized by phagocytes. Macrophages produce proinflammatory cytokines such as interleukin-1*β* (IL-1*β*) and tumor necrosis factor-*α* (TNF-*α*), thus enhancing an immune response [2]. Elevation of these signal molecules in plasma/serum may lead to alterations in lipid metabolism and cause hyperlipidemia [3]. In addition, in patients with diabetes mellitus (DM), periodontitis also adversely affects glycemic control [4].

DM results from insufficient insulin action (either absolute or relative). This most common metabolic disease is characterized by various degrees of chronic hyperglycemia, which together with an increased availability of free fatty acids is responsible for glucotoxicity and lipotoxicity in diabetes [5]. Apart from the systemic effects of diabetes, recent evidence suggests that local changes in the periodontal tissues are characterized by enhanced interactions between leukocytes and endothelial cells and altered leukocyte functions [6]. Increased levels of reactive oxygen species [7] and proinflammatory cytokines (IL-1*β*, IL-6, and TNF-*α*) lead to chronic low-grade inflammation, change of cytokine spectrum, and activation of innate immunity; alterations are present in both type 1 and type 2 diabetes mellitus (T1DM and T2DM, respectively) [8]. A meta-analysis by Chávarry et al. [9] identified T2DM as a moderate risk factor for CP, the effect of T1DM being less clear. It is probable that there is individual patient variability in the degree to which glycemic control influences periodontal status [10], and vice versa, periodontitis can contribute to the development of other diabetic complications, such as nephropathy (DN), retinopathy (DR), and neuropathy (DPN) [4].

IL-1*α* and IL-1*β* are produced during inflammation and induce production of many other cytokines, amplifying their proinflammatory action. They must be tightly controlled by endogenous inhibitors, such as IL-1 receptor antagonist (IL-1RA) and soluble IL-1 receptors, to prevent an acute and chronic overproduction of proinflammatory cytokines [11]. Polymorphisms in the *IL-1* cluster genes are located on chromosome 2q12 [12] and are composed of three ligands (*IL-1A*, *IL-1B*, and *IL-1RN*). To this date, only a few studies have focused on variability in the *IL-1* genes in diabetic patients with CP [13–17].

The first aim of this study was to investigate two single-nucleotide polymorphisms (SNPs) and one VNTR polymorphism in the *IL-1* gene cluster (*IL-1A* (−889C/T, rs1800587), *IL-1B* (+3953C/T, rs1143634), and *IL-1RN* (gene for IL-1 receptor antagonist, IL-1RA, 86 bp tandem repeats in intron 2)) in CP patients, diabetic patients of both types (T1DM and T2DM), and healthy controls in the Czech population. The second objective was to compare the subset of diabetic patients with CP and generally healthy patients without/with CP or diabetic patients according to their *IL-1* haplotype profile. Finally, assuming higher circulating IL-1*β* levels in diabetic patients with CP than in nondiabetic patients with CP, we, as the third aim, analyzed plasma levels in these groups of patients and their correlation to polymorphisms in the *IL-1* gene cluster. In addition, we examined IL-1*β* levels in mononuclear cells of generally healthy subjects after stimulation by oral pathogens, mitogens, or heat shock protein 70 (HSP70).

## 2. Materials and Methods

The study was performed with the approval of the Committees for Ethics of the Faculty of Medicine, Masaryk University, Brno (No. 13/2013), and St. Anne's Faculty Hospital. Written informed consent was obtained from all participants in line with the Declaration of Helsinki before inclusion in the study.

### 2.1. Subjects and Clinical Examinations

The cross-sectional study comprised a total of 1016 individuals, including 527 unrelated Caucasian subjects from the South Moravia region of the Czech Republic, with DM duration of at least 3 years. The diagnosis of T1DM (132 patients) or T2DM (395 patients) was originally based on the presence of clinical symptoms (such as polyuria, polydipsia, and weight loss) and biochemical parameters (glycemia, ketoacidosis, and autoantibody status in T1DM) in the outpatient unit of the diabetology centers in Brno. All patients were receiving antidiabetic drugs or insulin therapy and other medicaments according to the presence of diabetic complications, such as DN, DR, and DPN, and other comorbidities, as described in our cohort previously [18]. Levels of glycemia, glycated hemoglobin (HbA1c), total cholesterol, triglycerides, high-density lipoprotein (HDL), low-density lipoprotein (LDL), body mass index (BMI), and further parameters were recorded.

The periodontal status was evaluated in subgroups of the diabetic patients (38 T1DM and 89 T2DM patients), 225 healthy controls and 264 CP subjects recruited from the patient pool of the Clinic of Stomatology, St. Anne's Faculty Hospital, Brno, in the period of 2013–2017. The diagnosis of periodontitis/nonperiodontitis was based on the detailed clinical examination, medical and dental history, tooth mobility, and radiographic assessment [19]; the exclusion criteria for the studied cohort were described in our previous study [20].

### 2.2. Genetic Analysis

Genomic DNA was isolated from peripheral blood by a standard protocol and archived in the DNA bank at the Department of Pathophysiology, Faculty of Medicine, Masaryk University, Brno, Czech Republic. Polymorphisms *IL-1A* (−889C/T, rs1800587) and *IL-1B* (+3953C/T, rs1143634) were genotyped by polymerase chain reaction, with restriction analysis (PCR-RFLP) according to protocols published previously [21, 22]. The VNTR PCR method was used for the detection of 86 bp tandem repeats number in the second intron of the *IL-1RN* gene [23]. *IL-1RN* alleles were coded as *IL-1RN*^∗^1 (4 repeats, 412 bp), *IL-1RN*^∗^2 (2 repeats, 240 bp), *IL-1RN*^∗^3 (3 repeats, 326 bp), *IL-1RN*^∗^4 (5 repeats, 498 bp), *IL-1RN*^∗^5 (6 repeats, 584 bp), and *IL-1RN*^*∗*^6 allele (1 repeat, 154 bp). Details and modifications of PCR conditions were published in our previous study by Izakovicova Holla et al. [24].

### 2.3. Plasma Level Analysis

Levels of IL-1*β* in plasma were measured in 60 randomly selected subjects (20 CP, 20 T1DM + CP, and 20 T2DM + CP patients) at the Department of Biochemistry, Faculty of Medicine, Masaryk University, Brno. Plasma samples were prepared from venous blood, collected into a tube with EDTA (S-Monovette® 9 mL K3E, Sarstedt, Germany) by centrifugation (465 g/10 minutes/4°C), and stored at −70°C within 30 minutes after collection. For the determination of cytokine concentrations, Cytokine Human 10-Plex Panel for Luminex® Platform (Invitrogen™, California, USA), Luminex 200™ analyzer with xPONENT 3.1 Software (Luminex Corporation, USA), and Milliplex™ Analyst v 3.4 Software (VigeneTech, USA) were used.

In addition, examination of IL-1*β* levels in unstimulated and stimulated cells of 60 generally healthy subjects was performed in the laboratory of the Institute of Clinical and Experimental Dental Medicine, General University Hospital, and First Faculty of Medicine, Charles University, Prague. Isolation, cultivation, and stimulation of cells by selected periodontal bacteria (*A. actinomycetemcomitans*, *P. gingivalis*, *P. intermedia*, and *T. forsythia*), mitogens, or HSP70 were described previously [25]. IL-1*β* levels were determined in mononuclear cells isolated from 20 ml of heparinized blood using Fluorokine® MAP Human MultiAnalyte Profiling Base Kit, Luminex® 100™ analyzer, and Luminex 100 IS™ Software (R&D Systems, USA).

### 2.4. Statistical Analysis

Statistical analysis was performed using the statistical package Statistica v. 13 (StatSoft Inc., USA). Standard descriptive statistics were applied in the analysis: absolute and relative frequencies for categorical variables, and mean with standard deviation (SD) or median with quartiles for quantitative variables. To compare independent groups, one-way analysis of variance (ANOVA) and the Kruskal–Wallis ANOVA were performed to compare continuous variables. For exclusion of outlier values (IL-1*β* plasma levels), Grubb's test was used (*P* < 0.05). The allele frequencies were calculated from the observed numbers of genotypes. The differences in the allele frequencies were tested by Fisher's exact test; the Hardy–Weinberg equilibrium (HWE) and genotype frequencies were calculated by the chi-square test (*χ*^2^). To examine the linkage disequilibrium (LD) between polymorphisms, pairwise LD coefficients (D´) and haplotype frequencies were calculated using the SNP Analyzer 2 program (http://snp.istech.info/istech/board/login_form.jsp). The association was described by odds ratios (OR) with 95% confidence intervals (95%CI). Only the values of *P* less than 0.05 were considered as statistically significant.

## 3. Results

### 3.1. Clinical Data Analysis

The mean ages of the healthy controls and patient with CP did not differ significantly (*P* > 0.05); however, patients with T1DM were significantly younger (*P* < 0.05), and in contrast, patients with T2DM were significantly older than those in healthy controls (*P* < 0.01). There were no significant differences between the subjects with T1DM/T2DM and/or CP and the controls relating to the male/female ratio. The BMI values were the highest in the groups of T2DM and T2DM + CP, which differed statistically significantly from other groups (*P* < 0.05). All T1DM (*N*=38) and T2DM (*N*=89) patients who were examined at the Department of Periodontology were affected by periodontitis. The duration of DM in the patients with T1DM was approximately double than that of DM in T2DM patients. Slightly lower HbA1c levels in groups of patients with T1DM + CP and T2DM + CP compared with the group of all DM patients can be given by a higher interest of these patients in their health condition (only these patients accepted the offer to be examined by periodontist). The frequencies of diabetic complications (DN, DR, and DPN) in the whole diabetic cohort vs. the subgroup of T1DM or T2DM patients with CP are summarized in Table 1.

### 3.2. SNPs Analysis

The frequencies of *IL-1* genotypes were in HWE in the control subjects (*P* > 0.05, see Table 2). *IL-1RN*^∗^5 allele (6 repeats, 584 bp) was not detected in our sample. For calculation of allele frequencies and haplotype analysis, alleles with more than two repeat units in the *IL-1RN* gene were grouped together as *IL-1RN*^∗^L (“long” allele) and allele with 2 repeats was marked as *IL-1RN*^∗^S (“short” allele) [26]. As *IL-1RN*^∗^6 allele (with 1 repeat, 154 bp) found in one CP subject cannot be considered as typical “short” allele, this sample was excluded from both these analyses.

None association between *IL-1A* (−889C/T) SNP or a so-called “double genotype” (*IL-1A* (−889C/T)/*IL-1B* (+3953C/T)) and CP and/or DM was found, but the *IL-1B*^*∗*^T allele (+3953C/T) was less frequent in CP patients than in healthy controls (21.4% vs. 28.0%, *P* < 0.05, OR = 0.70, 95%CI = 0.52–0.94). Significant differences were found in the *IL-1RN*^∗^S allele frequencies and also in *IL-1RN*^∗^12 (vs. *IL-1RN*^∗^11) genotype frequencies between T1DM patients and healthy controls (*P* < 0.01, OR = 0.64, 95%CI = 0.44–0.92 and *P* < 0.01, OR = 0.46, 95%CI = 0.28–0.76). Comparison of the allele frequencies of *IL-1RN* in patients with T1DM vs. T2DM showed a significant difference between these groups (*P* < 0.05).

### 3.3. Haplotype Analysis

Combination of multiple SNPs sites showed eight *IL-1* haplotypes with frequency more than 1% (see Table 3). Pairwise linkage disequilibrium (LD) for all possible 2-way comparisons among 3 polymorphisms in the *IL-1* gene cluster in CP, T1DM, T2DM, and control groups was measured by Lewontin standardized disequilibrium coefficient (|D´|, data not presented).

The distribution of these haplotype frequencies between controls and patient groups showed significant differences. Haplotype T[*IL-1A*(−889C/T)]/T[*IL-1B* (+3953C/T)]/L[*IL-1RN*] was associated with lower risks for CP (*P* < 0.01, OR=0.64, 95% CI = 0.47–0.88), similar to haplotype CCS (*P* < 0.01, OR = 0.58, 95% CI = 0.39–0.87) with T1DM. On the contrary, CTL haplotype (*P* < 0.05, OR = 1.98, 95% CI = 1.01–3.87) was found in a higher frequency in T1DM patients vs. healthy subjects.

### 3.4. IL-1*β* Plasma Level Analysis

**Figure 1:**
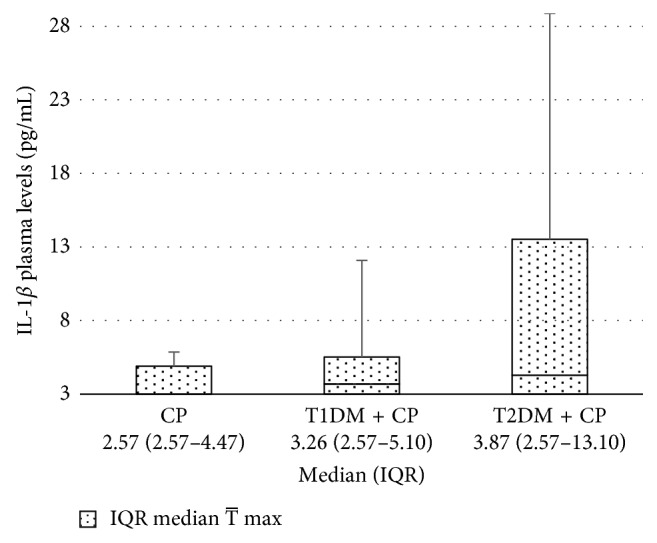
Comparison of IL-1*β* plasma levels in patients with CP (*N*=20), T1DM + CP (*N*=20), and T2DM + CP (*N*=20). CP = chronic periodontitis, T1DM = type 1 diabetes mellitus, T2DM = type 2 diabetes mellitus. For samples with values of IL-1*β* under detection limit (<2.58 pg/mL) were assigned a value of 2.57 pg/mL. For exclusion of outlier values, three CP and one T1DM + CP samples were used in Grubb's test (*P* < 0.05). The Mann–Whitney *U* test (two-tail) was used to calculate significant differences.

No significant differences in IL-1*β* plasma levels in diabetic patients and generally healthy subjects with similar periodontal conditions were found (see Figure 1). In twenty examined patients (nine CP, five T1DM + CP, and six T2DM + CP), IL-1*β* plasma levels were under the detection limit (<2.58 pg/mL); for these samples, a value of 2.57 pg/mL was assigned for the statistical analyses. IL-1*β* plasma levels in the whole studied set (*N*=60) were independent of the *IL-1B* or *IL-1RN* genotypes distribution (see Table 4); this result was also confirmed by the analysis of IL-1*β* levels in peripheral blood mononuclear cells (PBMCs) in generally healthy population (data not shown). However, high significant differences were observed in IL-1*β* levels between unstimulated PBMCs and cells after stimulation by Pokeweed mitogen (PWM), PWM in costimulation by Concavalin A (Con A), selected periodontal bacteria (*A. actinomycetemcomitans*, *P. intermedia*, and *T. forsythia*) (all *P* < 0.001), or HSP70 (*P* < 0.02, see Table 5).

## 4. Discussion

Diabetes and periodontitis are complex diseases with a bidirectional relationship [27]. Despite long-established evidence that hyperglycemia in diabetes is associated with adverse periodontal outcomes, the mechanism between these two states is not fully understood yet [10, 28]. Chronic inflammation, a common feature in the pathogenesis of both CP and DM, is related to the accumulation of activated innate immune cells in tissues, which results in the release of inflammatory mediators, such as IL-1 family cytokines. In this case-control study, we analyzed variability in the *IL-1* gene cluster and IL-1*β* plasma levels in patients with CP with/without diabetes in comparison to healthy controls. Minor allele frequency (MAF) of *IL-1A* (−889C/T) found in our healthy controls was 31%, which is in line with the *IL-1A*^∗^T allele frequency according to NCBI database (28%) (http://www.ncbi.nlm.nih.gov/projects/SNP/snp_ref.cgi?rs=1800587), whereas in healthy European subjects, higher MAF was reported (37% [29]; 56%) [30]. The MAF of *IL-1B* (+3953C/T, rs1143634) in the Czech population (28%) was similar as *IL-1B*^∗^T allele frequency in healthy European subjects (from 24% to 29%) [29–32], whereas the NCBI database provides MAF of only 13% (http://www.ncbi.nlm.nih.gov/projects/SNP/snp_ref.cgi?rs=1143634). *IL-1RN*^∗^S allele in European population varies from 21% to 31% [33–36], which is consistent with that in our observation (27%).

### 4.1. Chronic Periodontitis

In the context of CP, variability in the *IL-1* gene cluster has been investigated many times with conflicting results. To our knowledge, no study has examined the relationship between specific allele combinations of these three *IL-1* gene variants (*IL-1A* (−889C/T), *IL-1B* (+3953C/T), and *IL-1RN* (VNTR)) and periodontal diseases. In our population, haplotype TTL seems to be protective against the development of CP and the *IL-1B*^∗^T allele is of the same importance. This observation supports our previously detected association between *IL-1B*^∗^C allele and a higher risk for CP [37]. Nevertheless, da Silva et al. [38] came to opposite conclusions in their meta-analysis including 54 studies in different populations, which associated T allele in Caucasian carriers with a 1.25-time higher risk of developing CP than C allele carriers. In addition, no association between *IL-1A* (−889C/T) polymorphism and CP in the Czech population was found; this is in contrast to a recent meta-analysis by da Silva et al. [39]. In line with a meta-analysis by Ding et al. [40], we found no differences in allele or genotype frequencies in *IL-1RN* variants between CP patients and healthy controls.

We suggest that these discrepancies can be caused not only by population differences in allele frequencies but also by an interaction effect of *IL-1* genes variants. According to Morris and Kaplan [41], haplotype-based analysis can be more useful than an analysis based on individual polymorphisms in complex multifactorial diseases, as confirmed by our results.

Screening of SNPs and genome-wide studies has yielded new genetic information without a definitive solution for the management of periodontal disease [42]. However, SNP variations are no longer sufficient for establishing a relationship with periodontal disease. From this reason, the analyses of differential gene expression, performed using high-throughput experimental methods, such as microarray analysis, should be used in the future research [43]. Thus, the genetic basis of periodontal disease is moving from experimental evidence to a more consistent translation effect on diagnosis and development of new strategies to modulate the host [42].

### 4.2. T1DM

In our study, a significant association between “short” variant in intron 2 of the *IL-1RN* gene and lower risk of developing T1DM was observed (*P* < 0.01). Our findings are in line with the study in Egyptian population, where this minor allele and *IL-1RN* SS genotype were also less prevalent in patients with T1DM than in healthy controls [44]. On the other hand, in the recent study by Ali et al. [45], *IL-1RN*^∗^S allele and *IL-1RN* 12 genotype were present more frequently in the Saudi children with T1DM than healthy controls. *IL-1RN*^∗^S allele was previously associated with increased production of IL-1*β* [46] and IL-1RA, and also with reduced production of IL-1*α* by normal monocytes [47]. Thus, not only environmental factors as reported by Cullup et al. [48] but also variability in a number of 86 bp tandem repeats in intron 2 of the *IL-1RN* gene may be related to an imbalance between IL-1 anti- and proinflammatory protein levels.

In correlation to *IL-1* gene polymorphism, Krikovszky et al. [49] described the *IL-1B*^∗^T allele as risk in Hungarian children with T1DM. Although no significant relationships between *IL-1A* (−889C/T) or *IL-1B* (+3953C/T) variants and T1DM in Czech patients were found, the *IL-1B*^∗^T allele in combination with *IL-1A*^∗^C and *IL-1RN*^∗^L alleles was associated with an increased risk of T1DM.

Our results suggest that variability in the *IL-1* gene cluster, especially in the gene for IL-1RA, may be one of the factors in the pathogenesis of T1DM in Czech patients. This conclusion is supported by results from the haplotype analysis as the opposing haplotypes in *IL-1* CCS vs. CTL were significantly associated with lower vs. higher risk of T1DM.

As we supposed, variability in the *IL-1* gene cluster may play an important role in the pathogenesis of both diseases, and frequencies of the three most represented haplotypes (CCL, CCS, and TTL) in the T1DM + CP subjects seem to be intermediate between frequencies of CP and T1DM patients. Although a similar prevalence of CTL haplotype was found in the T1DM + CP subgroup and T1DM group, there was no statistical significance due to a low number of the examined type 1 diabetic patients with CP (*N*=38).

### 4.3. T2DM

No significant differences in *IL-1A*, *IL-1B*, or *IL-1RN* gene variability between healthy controls and T2DM Czech patients with/without CP were found. Independently of periodontal status, Luotola et al. [50] suggested gender-specific associations between *IL-1A* (-899C/T) and *IL-1B* (+3953C/T) gene variants and a higher risk of T2DM, especially in men. Previously, the same authors described a relationship between *IL-1B*^∗^T allele (+3953C/T) and higher blood glucose levels in Finnish patients with DM [51]. In Malayalam-speaking Dravidian population, differences in *IL-1B*^∗^T allele and *IL-1B* TT genotype distribution between T2DM + CP (*N*=51) and CP patients were found [16]. *IL-1RN^*∗*^*S allele and *IL-1RN* SS genotype were also associated with risk of developing T2DM in the Indian population [52, 53].

Our results correspond to observations in Chilean population focused on the same polymorphism in the *IL-1* gene cluster in T2DM and/or CP patients [15]. Deppe et al. [17] in their recent study assumed that CP in T2DM patients was most strongly associated with inadequate oral hygiene, whereas variability in the *IL-1* genes and differences in oral microbiota seemed to play a subordinate role. Furthermore, Guzman et al. [13] found only a borderline association between *IL-1B* (+3953C/T) SNP and the incidence of periodontal disease in Caribbean diabetic population. Struch et al. [14] reported that diabetic carriers of T allele in “double genotype” *IL-1A*/*IL-1B* had an enhanced risk for periodontal disease in comparison with their *IL-1* wild-type counterparts. Although the sample sizes in these three German studies (*N*=66 [13] or 69 [14] or 38 patients with T2DM + CP [17]) are comparable with the size of our cohort (*N*=89), Czech T2DM patients were selected from ethnically homogenous population, and in addition, other 306 T2DM patients with unknown periodontal status were included.

Results of haplotype analysis showed an interesting trend: the CCL, CCS, and TTL haplotype frequencies in the diabetic patients with CP were the highest or the lowest among CP, T2DM, and T2DM + CP patient groups. Although the TTL haplotype was considered protective for the development of CP in our population, frequency of this allele combination in T2DM + CP patients was closer to the results in healthy controls.

### 4.4. IL-1*β* Plasma Levels

IL-1*β* levels in gingival crevicular fluid (GCF) of patients with CP are significantly higher than those in patients with gingivitis and periodontally healthy individuals [54–56]. Therefore, elevated IL-1*β* GCF levels, but not plasma levels, were suggested as reliable inflammatory biomarkers in periodontal diseases [57]. In T1DM patients, increased levels of IL-1*β* in GCF were also found [58], and according to Aspriello et al. [59], IL-1*β* levels in T1DM patients with periodontitis are affected by the duration of DM. Meta-analysis by Atieh et al. [60] presented significant differences in IL-1*β* GCF levels between T2DM with CP patients and nondiabetic controls with the similar periodontal conditions.

Only a few studies reported inconsistent results for IL-1*β* concentrations in plasma/serum in relation to periodontal diseases or DM. Levels of circulating IL-1*β* were elevated in T1DM children than the control group [61, 62] and mRNA levels of IL-1*β* in peripheral blood leukocytes were found higher in T2DM patients than in healthy controls [63]. In another study, Sapathy et al. [64] suggested that changes of serum IL-1*β* levels were influenced by abdominal obesity and periodontal status independently even in the absence of DM and smoking.

In our study, the IL-1*β* plasma levels were under the detection limit in nine of 20 patients with CP. However, low values of this proinflammatory cytokine are not surprising because these patients are in good general health. Previously, Orozco et al. [54] also demonstrated zero concentration of IL-1*β* in the serum samples of both periodontitis and gingivitis patients. Nevertheless, Gümüş et al. [65] calculated median 11.7 pg/mL of serum IL-1*β* levels in their CP population. The higher circulating levels of IL-1*β* in 55% of Czech CP subjects may be the consequence of their smoking status since other risk factors linked with the increase in proinflammatory cytokines were minimized by strict criteria for inclusion in the study.

To our knowledge, this is the first study comparing IL-1*β* plasma levels in T2DM patients with CP and nondiabetic patients with CP. Although we assumed significant higher concentrations of this cytokine in all diabetic patients than in generally healthy patients with similar periodontal conditions, only a slightly elevated IL-1*β* levels were found in T1DM + CP or T2DM + CP patients compared with the CP subjects (median 3.26 pg/mL or 3.87 pg/mL vs. 2.57 pg/mL).

Furthermore, Santtila et al. [46] published that mononuclear cells from noncarriers of *IL-1B^∗^*T allele (+3953C/T) had a slight, but nonsignificantly, elevated capacity to produce IL-1*β in vitro*. Our results are in line with this finding; the single variants of *IL-1B* or *IL-1RN* polymorphisms are not crucial in the protein production as apparent from both analyses of levels in plasma or in PBMCs. In our study, IL-1*β* levels in mononuclear cells of 60 generally healthy subjects after stimulation by periodontal bacteria (except *P. gingivalis*), mitogens, or HSP70 were examined. The obtained data confirmed our premise that these stressors significantly affect the production of proinflammatory IL-1*β*.

The main limitation of this study is the fact that only subgroups of diabetic patients with T1DM or T2DM were checked for their periodontal status as the number of patients willing to be examined at the Periodontology Department was low. On the other hand, the number of T2DM + CP patients is still higher than in previous studies in other populations. Besides the size of the overall study cohort (*N*=1016), a further positive aspect of this study is a haplotype approach applied which presents a more complex view on the variation in the *IL-1* gene cluster. We assume that the discrepancy between our results and that from recent meta-analyses focusing on the relation between *IL-1* polymorphisms and CP can be partly caused by the fact that these meta-analyses studied only single variants in the *IL-1A* [38] or *IL-1B* genes [39] and by publication “bias” when negative results are not published.

## 5. Conclusions

In conclusion, results of the *IL-1* gene cluster analysis suggest that variability especially in the *IL-1B* and *IL-1RN* genes may be one of the factors in the susceptibility to T1DM and CP, although the single variants of these polymorphisms are not crucial for the protein production. In addition, no differences in IL-1*β* plasma levels were found between Czech diabetic patients with CP and generally healthy subjects with similar periodontal conditions.

## Figures and Tables

**Table 1:** Demographic data of the studied subjects with CP, T1DM (and T1DM + CP subgroup), T2DM (and T2DM + CP subgroup), and healthy controls.

Characteristics	Controls (*N*=225)	CP (*N*=264)	T1DM (*N*=132)	T1DM + CP (*N*=38)	T2DM (*N*=395)	T2DM + CP (*N*=89)
Age (mean years ± SD)	53.9 ± 11.0	56.1 ± 8.2	45.8 ± 14.2^*∗*^	49.4 ± 10.9	67.5 ± 10.6^*∗*^	67.3 ± 10.0^*∗*^
Gender (males/females)	114/111	122/142	68/64	16/22	200/195	45/44
Smoking (no/yes, %)	72.9/27.1	71.6/28.4	—	76.3/23.7	—	87.7/12.3
BMI (mean ± SD)	23.2 ± 4.6	26.8 ± 4.0	24.5 ± 6.1	25.3 ± 3.1	28.6 ± 10.0^*∗*^	30.3 ± 6.2^*∗*^
Duration of DM (mean years ± SD)	n.a.	n.a.	21.5 ± 9.8	24.0 ± 10.7	15.1 ± 8.8	11.0 ± 8.7
HbA1c (mmol/mol, mean ± SD)	n.a.	n.a.	77.1 ± 17.3	69.8 ± 11.8	72.6 ± 23.9	58.0 ± 15.6
DN (no/yes, %)	n.a.	n.a.	43.6/56.4	71.0/29.0	7.6/92.4	80.2/19.8
DR (no/yes, %)	n.a.	n.a.	24.7/75.3	40.6/59.4	52.9/47.1	92.4/7.6
DPN (no/yes, %)	n.a.	n.a.	36.7/63.3	47.1/52.9	49.3/50.7	84.4/15.6

CP = chronic periodontitis; *N* = number of subjects; SD = standard deviation; T1DM = type 1 diabetes mellitus; T2DM = type 2 diabetes mellitus; BMI = body mass index; DN = diabetic nephropathy; DPN = diabetic peripheral neuropathy; DR = diabetic retinopathy; HbA1c = glycated hemoglobin; — = unknown in diabetic patients without CP; n.a. = nonapplicable. ^*∗*^*P* < 0.05 in comparison to healthy controls (evaluated by the Kruskal–Wallis ANOVA test).

**Table 2:** *IL-1* genotype and allele frequencies in patients with CP, T1DM (and T1DM + CP subgroup), T2DM (and T2DM + CP subgroup), and healthy controls.

Genotypes alleles	Controls (*N*=225)	CP (*N*=264)	OR (95% CI)	T1DM (*N*=132)	OR (95% CI)	T1DM + CP (*N*=38)	OR (95% CI)	T2DM (*N*=395)	OR (95%CI)	T2DM + CP (*N*=89)	OR (95% CI)
*IL-1A* (−889C/T)	*N* (%)	*N* (%)		*N* (%)		*N* (%)		*N* (%)		*N* (%)	
CC	105 (46.7)	145 (54.9)	1.00	61 (46.2)	1.00	20 (52.6)	1.00	198(50.1)	1.00	46 (51.7)	1.00
CT	102 (45.3)	104 (39.4)	0.74 (0.51–1.07)	62 (47.0)	1.05 (0.67–1.63)	15 (39.5)	0.77 (0.37–1.59)	160 (40.5)	0.83 (0.59–1.17)	34 (38.2)	0.76 (0.45–1.28)
TT	18 (8.0)	15 (5.7)	0.70 (0.34–1.36)	9 (6.8)	0.86 (0.36–2.03)	3 (7.9)	0.88 (0.24–3.25)	37 (9.4)	1.09 (0.59–2.01)	9 (10.1)	1.14 (0.48–2.73)
C allele	312 (69.3)	394 (74.6)	1.00	184 (69.7)	1.00	55 (72,4)	1.00	556 (70.4)	1.00	126 (70.8)	1.00
T allele	138 (30.7)	134 (25.4)	0.77 (0.58–1.02)	80 (30.3)	0.98 (0.71–1.37)	21 (27.6)	0.86 (0.50–1.48)	234 (29.6)	0.95 (0.74–1.22)	52 (29.2)	0.93 (0.64–1.36)

*IL-1B* (+3953C/T)											
CC	115 (51.1)	164 (62.1)	1.00	73 (55.3)	1.00	18 (47.4)	1.00	220 (55.7)	1.00	50 (56.2)	1.00
CT	94 (41.8)	87 (33.0)	0.65(0.45–0.95)	48 (36.4)	0.80 (0.51–1.27)	17 (44.7)	1.16 (0.56–2.37)	143 (36.2)	0.80 (0.56–1.12)	30 (33.7)	0.73 (0.43–1.25)
TT	16 (7.1)	13 (4.9)	0.57 (0.26–1.23)	11 (8.3)	1.08 (0.48–2.46)	3 (7.9)	1.20 (0.32–4.53)	32 (8.1)	1.05 (0.55–1.98)	9 (10.1)	1.29 (0.54–3.12)
C allele	324 (72.0)	415 (78.6)	1.00	194 (73.5)	1.00	53 (69.7)	1.00	584 (73.8)	1.00	130 (73.0)	1.00
T allele	126 (28.0)	113 (21.4)^*∗*^	0.70 (0.52–0.94)	70 (26.5)	0.93 (0.66–1.31)	23 (30.3)	1.12 (0.66–1.90)	206 (26.2)	0.91 (0.70–1.18)	48 (27.0)	0.95 (0.64–1.40)

*IL-1RN* (VNTR)^§^											
11	108 (48.0)	121 (45.8)	1.00	83 (62.9)	1.00	19 (50.0)	1.00	220 (55.7)	1.00	48 (53.9)	1.00
12	87 (38.7)	110 (41.7)	1.13 (0.77–1.65)	31 (23.5)^*∗*^	0.46 (0.28–0.76)	11 (28.9)	0.72 (0.32–1.59)	129 (32.7)	0.73 (0.51–1.04)	25 (28.1)	0.65 (0.37–1.13)
13	8 (3.6)	1 (0.4)	0.11 (0.01–0.91)	1 (0.8)	0.16 (0.02–1.33)	1 (2.6)	0.71 (0.08–6.01)	3 (0.8)	0.18 (0.05–0.71)	1 (1.1)	0.28 (0.03–2.31)
14	3 (1.3)	8 (3.0)	2.38 (0.62–9.20)	4 (3.0)	1.73 (0.38–7.96)	1 (2.6)	1.89 (0.19–19.19)	9 (2.3)	1.47 (0.39–5.55	4 (4.5)	3.00 (0.65–13.93)
22	17 (7.6)	17 (6.4)	0.89 (0.43–1.83)	8 (6.1)	0.61 (0.25–1.49)	2 (5.3)	0.67 (0.14–3.13)	31 (7.8)	0.90 (0.47–1.69)	9 (10.1)	1.19 (0.50–2.86)
23	0	1 (0.4)	#	1 (0.8)	#	1 (2.6)	#	0	#	0	#
24	2 (0.9)	5 (1.9)	2.23 (0.42–11.74)	3 (2.3)	1.95 (0.32–11.95)	2 (5.3)	5.68 (0.75–42.84)	3 (0.8)	0.74 (0.12–4.47)	2 (2.2)	1.38 (0.19–10.04)
44	0	0	#	1 (0.8)	#	1 (2.6)	#	0	#	0	#
16	0	1 (0.4) ^§§^	#	0	#	0	#	0	#	0	#
L allele	327 (72.7)	376 (71.5)	1.00	213 (80.7)	1.00	58 (76.3)	1.00	596 (75.4)	1.00	133 (74.7)	1.00
S allele	123 (27.3)	150 (28.5)	1.06 (0.80–1.40)	51 (19.3)^*∗*^	0.64 (0.44–0.92)	18 (23.7)	0.83 (0.47–1.46)	194 (24.6)	0.87 (0.67–1.13)	45 (25.3)	0.90 (0.61–1.34)

CI = confidence interval; CP = chronic periodontitis; OR = odds ratio; T1DM = type 1 diabetes mellitus; T2DM = type 2 diabetes mellitus. ^*∗*^*P* < 0.05 in comparison to healthy controls (evaluated by Fisher's exact test, without correction for multiple comparisons). ^#^Cannot be assessed because of the small number. ^§^Alleles with more than two repeat units were grouped together as *IL-1RN* ^∗^ L (“long” allele) and 2 repeat units were marked as *IL-1RN*^*∗*^S (“short” allele). ^§§^Sample with rare genotype *IL-1RN*16 was excluded from allele frequencies and haplotype analysis.

**Table 3:** Estimated frequencies (%) of *IL-1* haplotypes in patients with CP, T1DM (and T1DM + CP subgroup), T2DM (and T2DM + CP subgroup), and healthy controls.

*IL-1A* (−889C/T)	*IL-1B* (+3953C/T)	*IL-1RN* (VNTR)^§^	Controls (*N*=225)	CP (*N*=264)	T1DM (*N*=132)	T1DM + CP (*N*=38)	T2DM (*N*=395)	T2DM + CP (*N*=89)
C	C	L	43.14	48.66	47.47	46.60	46.14	49.37
C	C	S	21.64	21.50	14.03^*∗*^	17.39	18.11	17.86
T	T	L	21.35	13.35^*∗*^	15.62	18.03	17.42	19.06
T	C	L	4.99	5.83	9.40	3.31	6.77	3.65
C	T	L	3.19	3.65	8.20^*∗*^	8.38	5.12	2.65
T	T	S	2.23	2.53	2.70	3.85	2.79	2.16
T	C	S	2.10	3.76	2.59	2.44	2.65	4.35
C	T	S	1.36	0.73	—	—	1.02	0.91

CP = chronic periodontitis; T1DM = type 1 diabetes mellitus; T2DM = type 2 diabetes mellitus. ^§^For haplotype analysis, alleles with more than two repeat units were grouped together as *IL-1RN*^*∗*^L (“long” allele) and 2 repeat units were marked as *IL-1RN*^*∗*^S (“short” allele). Haplotypes are ordered according to decreasing haplotype frequency in the healthy control subjects. ^*∗*^*P* < 0.05 in comparison to healthy controls (without correction for multiple comparisons).

**Table 4:** IL-1*β* plasma levels and polymorphisms in *IL-1B* and *IL-1RN* genes.

Genotypes	All groups (*N*=60)	IL-1*β* levels (pg/mL), median (IQR)
*IL-1B* (+3953C/T)		
CC	31 (51.7)	3.26 (2.57–5.43)
CT	26 (43.3)	3.26 (2.57–10.59)
TT	3 (5.0)	2.69 (2.57–9.88)

*IL-1RN* (VNTR)^§^		
LL	37 (61.7)	3.26 (2.57–10.24)
LS	17 (28.3)	3.85 (2.57–9.35)
SS	5 (8.3)	3.26 (2.63–6.79)

IQR = interquartile range. ^§^Alleles with more than two repeat units were grouped together as *IL-1RN*^*∗*^L (“long” allele) and 2 repeat units were marked as *IL-1RN*^*∗*^S (“short” allele); genotypes are known only in 59 subjects. The Kruskal–Wallis ANOVA test was used to calculate significant differences.

**Table 5:** Differences in IL-1*β* levels between unstimulated and stimulated PBMCs (*N*=60).

PBMCs	IL-1*β* levels (pg/mL), median (IQR)	*P* value
Stimulated by		
PWM	481.97 (100.89–938.66)	<0.001
PWM + ConA	533.30 (141.45–950.37)	<0.001
HSP70	95.28 (23.36–371.60)	0.02
*A. actinomycetemcomitans*	483.93 (162.48–890.50)	<0.001
*P. gingivalis*	27.88 (6.47–118.14)	0.34
*P. intermedia*	249.38 (57.83–441.80)	<0.001
*T. forsythia*	201.08 (44.39–583.32)	<0.001
Unstimulated	52.55 (14.90–153.54)	—

CI = confidence interval; ConA = Concavalin A; HSP 70 = heat shock protein 70; OR = odds ratio; PBMCs = peripheral blood mononuclear cells; PWM = Pokeweed mitogen. The Wilcoxon matched pair test was used to calculate the significant difference.

## Data Availability

The clinical and genetic data used to support the findings of this study are restricted by the Committees for Ethics of the Faculty of Medicine, Masaryk University, Brno (No. 13/2013), in order to protect patient privacy. Data are available from the corresponding author via mail for researchers who meet the criteria for access to confidential data.
